# Large-scale production of megakaryocytes in microcarrier-supported stirred suspension bioreactors

**DOI:** 10.1038/s41598-018-28459-x

**Published:** 2018-07-05

**Authors:** Dorothee Eicke, Anja Baigger, Kai Schulze, Sharissa L. Latham, Caroline Halloin, Robert Zweigerdt, Carlos A. Guzman, Rainer Blasczyk, Constança Figueiredo

**Affiliations:** 1 Institute for Transfusion Medicine, Hannover Medical School, Hannover, 30625 Germany; 2 Department of Vaccinology and Applied Microbiology, Helmholtz Centre for Infection Research, Braunschweig, 38124 Germany; 3 Institute for Biophysical Chemistry, Hannover Medical School, Hannover, 30625 Germany; 4 Leibniz Research Laboratories for Biotechnology and Artificial Organs (LEBAO), Hannover, 30625 Germany; 5 REBIRTH Cluster of Excellence, Hannover Medical School, Hannover, 30625 Germany

## Abstract

Megakaryocytes (MKs) are the precursors of platelets (PLTs) and may be used for PLT production *in vivo* or *in vitro*, as well as a source for PLT-derived growth factors. Induced pluripotent stem cells represent an unlimited cell source for the *in vitro* production of MKs. This study aimed at developing an effective, xeno-free and scalable system to produce high numbers of MKs. In particular, microcarrier beads-assisted stirred bioreactors were evaluated as a means of improving MK yields. This method resulted in the production of 18.7 × 10^7^ MKs per 50 ml medium. Laminin-coated microcarriers increased MK production per iPSC by up to 10-fold. MKs obtained in this system showed typical features of mature MKs and were able to produce PLTs *in vitro* and *in vivo*. To increase safety, MKs produced in the bioreactors were irradiated; a procedure that did not affect their capability to form proPLTs and PTLs after transfusion. *In vitro* generated MKs represent a promising alternative to donor PLTs and open the possibility for the development of innovative MK-based cell therapies.

## Introduction

Megakaryocytes (MKs) are a rare cell type found in bone marrow. They not only play an essential role in the generation of platelets (PLTs), but also secrete a wide range of growth factors involved in the regulation of different mechanisms, such as tissue remodeling. Platelet (PLT) transfusion is required to prevent severe bleeding complications in patients suffering from thrombocytopenia. As donor-derived PLTs have a short shelf life, are limited by insufficient donor numbers and have an increased risk of bacterial contamination and deterioration caused by storage conditions^1,2^, it is highly desirable to develop alternative strategies. *In vitro* generated megakaryocytes (MKs) represent not only PLT precursor cells, but are themselves investigated as direct surrogates for PLTs in transfusion medicine. The first clinical studies assessing CD34^+^-derived MK progenitors after high dose chemotherapy showed promising results regarding the reduced need for supplementary PLT transfusion and demonstrated long-term safety of this approach^3–5^. In recent years, induced pluripotent stem cells (iPSCs) have gained plenty of interest in the cell therapy field as they constitute a virtually unlimited cell source and are associated with low ethical concern^6^. To produce MK and PLTs in quantities that meet the clinical need and of a quality that is in compliance with good manufacturing practice (GMP), a scalable system for iPSC culture and differentiation under serum- and xeno-free conditions has to be developed. While a number of bioreactor (BR) solutions for the large-scale production of PLTs from MKs derived from different cell sources have been published in recent years^7–11^, the feasibility of producing MKs in large-scale has not shown yet^12–14^. Well established 2D culture systems are not suitable to fulfil this demand due to their restricted surface to volume ratio, the amount of time required for manual passaging, inherent variability between setups, limited potential for online control of cultivation parameters and low cell yields, despite the development of stacked systems^15–18^. Suspension culture in stirred BRs represents the most favorable culture method for large-scale production in terms of scalability, simple design, straightforward handling (feeding, harvesting), control of cell density and distribution, online monitoring and control of culture conditions (pH, temperature, dO_2_, dCO_2_, agitation), and the homogeneous distribution of nutrients^16,17,19,20^. The common use of stirred suspension BRs by the biotechnology field for the production of antibodies and vaccines demonstrates their industrial value and will facilitate research translation from laboratory to commercial production settings^18^. To date, suspension culture setups including cell-only aggregates (cell-OAs)^21–23^, microcarrier (MC) culture^24,25^, and encapsulation^26,27^ have shown encouraging results for the expansion and differentiation of human pluripotent stem cells (hPSCs) into different specific cell types.

This study aimed at developing a scalable and efficient process to provide large amounts of MKs under serum- and xeno-free conditions. Aggregate- and in particular MC-based culture strategies in stirred suspension spinner flasks were tested to meet these requirements.

## Results

### Production of iPSC-derived MKs in stirred spinner flasks

**Figure 1:**
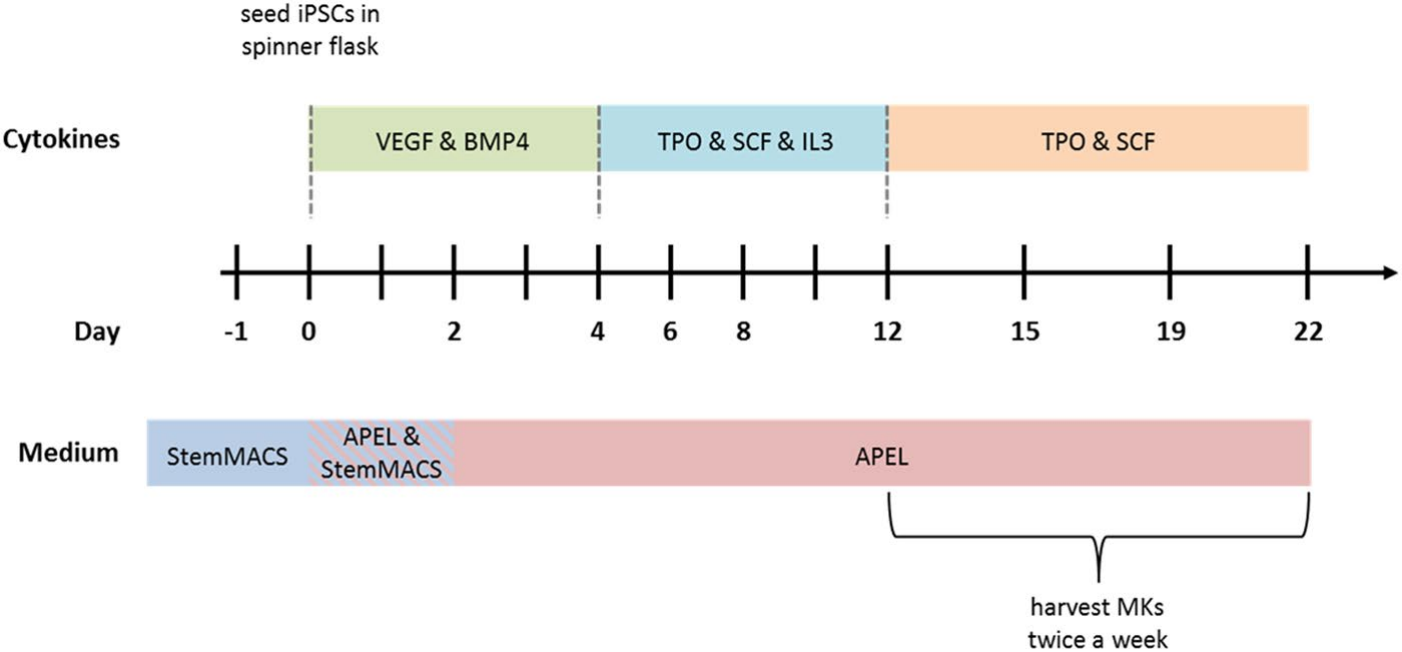
Schematic representation of the MK differentiation protocol from iPSCs. The scheme depicts the time sequence in which media and cytokine cocktails were used throughout the differentiation of iPSCs into MKs.

Medium and cytokine cocktails used to differentiate MKs in stirred bioreactors are consistent with those used for the differentiation of MKs from iPSCs, as we have described previously^28^ (Fig. 1).

**Figure 2:**
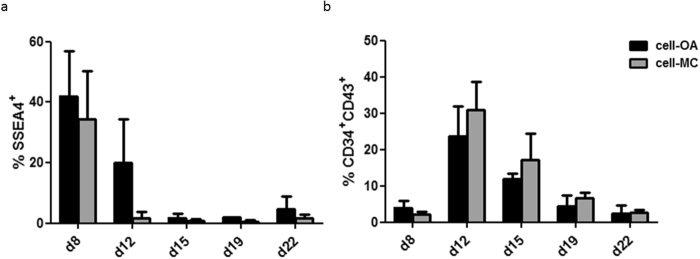
Kinetics of expression of pluripotency and progenitor markers during differentiation. The cells harvested from the differentiation supernatant in the spinner flask bioreactors were analyzed by flow cytometry for the pluripotency marker SSEA4 (**a**) and markers for hematopoietic progenitor cells, CD34 and CD43 (**b**) from day 8 until day 22. Graphs are depicted as mean ± SD of n ≥ 3.

Cells were harvested from the supernatant of cell-OAs or cell-MC aggregates twice a week starting from day 8 and were further cultivated in suspension flasks starting from day 12. Expression of the pluripotency marker SSEA4 decreased from 43.2 ± 12.5% in cell-OA differentiation and 34.4 ± 15.9% in cell-MC differentiation on day 8 to 2.8 ± 2.2% and 0.8 ± 0.6 on day 15, respectively (Fig. 2a). On day 19 the SSEA4 frequency was significantly lower in the cell-MC approach compared to cell-OA differentiation (p = 0.0286). Conversely, frequencies of hematopoietic progenitor cells co-expressing CD34 and CD43 increased from 4.0 ± 1.84% on day 8 to 23.7 ± 8.2% on day 12 and subsequently decreased to 2.3 ± 2.4% on day 22 in cell-OA differentiation (Fig. 2b). In cell-MC differentiations, CD34 and CD43 co-expression increased from 2.1 ± 0.9% on day 8 to 30.8 ± 7.8% on day 12 and following decreased to 2.6 ± 0.7 on day 22 (Fig. 2b).

**Figure 3:**
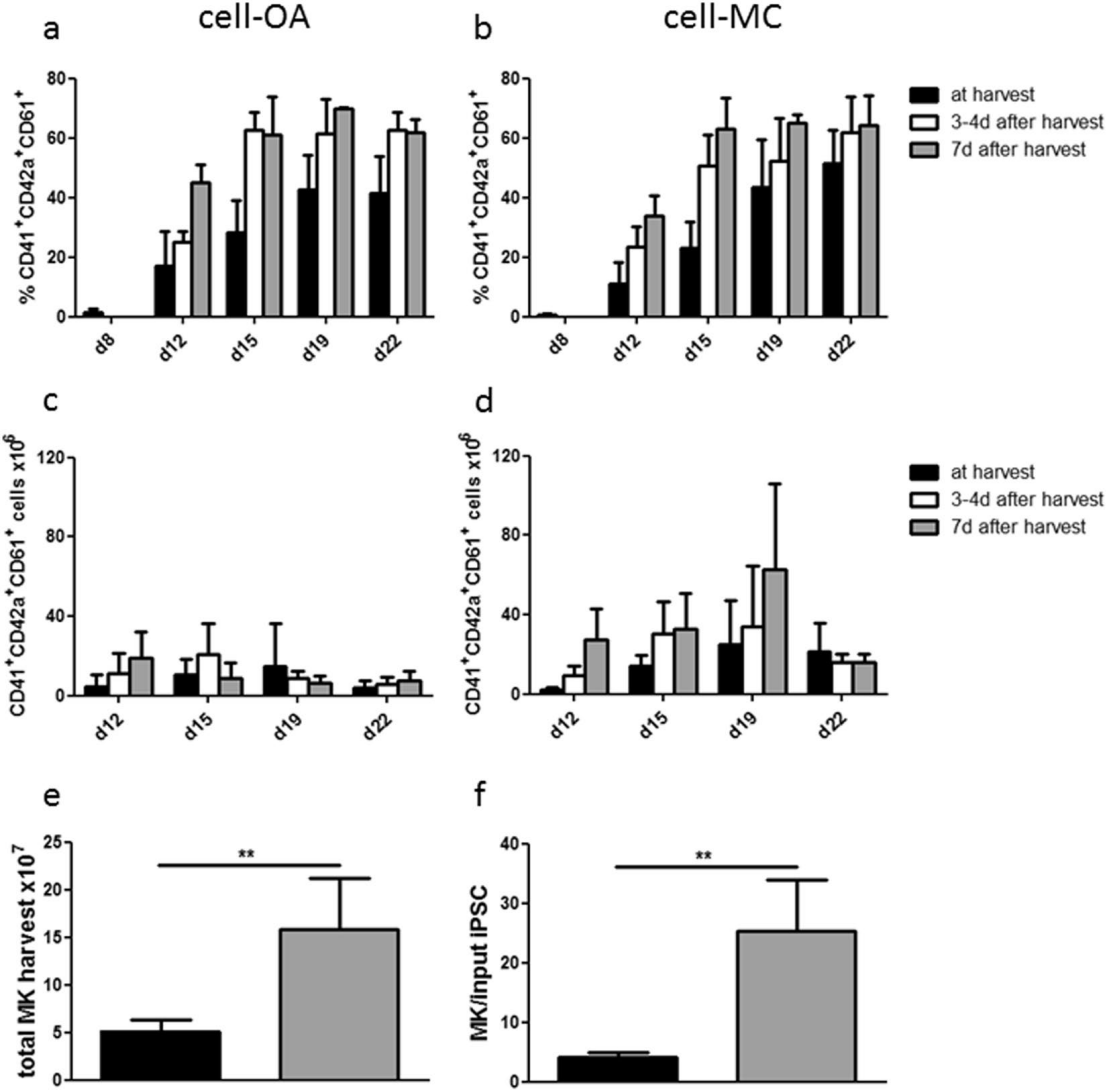
Characterization of phenotype and quantity of MKs differentiated in stirred flasks. Phenotype of the cells harvested from the differentiation from day 8 to day 22 was analyzed on the day of harvesting, after 3–4 days of subsequent cultivation, and after 7 days of further cultivation. Results are shown as the percentage of CD41^+^CD42a^+^CD61^+^ events from the cell-OA aggregate differentiation (**a**) and cell-MC aggregate differentiation (**b**). The numbers of triple positive MKs was calculated from day 12 to day 22 at the time of harvest, after 3–4 days and after 7 days from the cell-OA differentiation (**c**) and the cell-MC differentiation (**d**). The total quantity of harvested triple positive MKs calculated by the total sum of MK numbers from day 12, day 15, day 19, and day 22, either measured on the day of harvest or on 3–4 days or 7 days after harvest is depicted as 10^7^ MKs per spinner flask bioreactor (**e**) and the number of MKs per input iPSC (**f**). Graphs are depicted as mean ± SD of n ≤ 7.

Efficiencies of MK differentiation were analyzed by the co-expression of typical MK surface markers CD41, CD42a, and CD61. Frequencies of MKs in the supernatant increased until day 22, to 41.9 ± 11.5% CD41^+^CD42a^+^CD61^+^ cells in cell-OA differentiation and 51.3 ± 11.5% in cell-MC aggregate differentiation (Fig. 3a,b). Cells harvested from the supernatants in the bioreactors were transferred to suspension flasks and further cultivated for 7 days (Fig. 3a,b). CD41^+^CD42a^+^CD61^+^ cell fractions increased from 1.2 to 3.5 fold and 1.3 to 4.3 fold in cell-OA and cell-MC aggregate differentiations, respectively. The number of CD41^+^CD42a^+^CD61^+^ cells harvested from cell-OA differentiation increased from 4.6 ± 6.0 × 10^6^ on day 12 to 14.5 ± 21.8 × 10^6^ on day 19 and decreased to 4.3 ± 3.1 × 10^6^ on day 22 (Fig. 3c). The number of CD41^+^CD42a^+^CD61^+^ cells harvested on day 12 increased 2.5 fold and 4.1 fold after 3–4 days and after 7 days, respectively. Whilst the cell-OA differentiation harvest from day 15 increased initially but decreased until day 7 of continued cultivation, the harvest from day 19 decreases (0.4 fold) and from day 22 increases slightly (1.7 fold, Fig. 3c). The yield of CD41^+^CD42a^+^CD61^+^ cells from the cell-MC differentiation increased from 2.3 ± 1.4 × 10^6^ on day 12 to 24.8 ± 22.1 × 10^6^ on day 19 and decreased slightly to 21.3 ± 14.5 × 10^6^ on day 22 (Fig. 3d). Throughout the following 7 days of cultivation, the number of CD41^+^CD42a^+^CD61^+^ cells harvested from the cell-MC spinner flask on day 12 increased strongly (12.2 fold). Also, the cell harvest from day 15 and day 19 increased 2.4 fold and 2.5 fold, respectively. CD41^+^CD42a^+^CD61^+^ cell numbers harvested from the MC-based differentiation on day 22 decreased slightly (0.8 fold, Fig. 3d). In total 4.9 ± 1.3 × 10^7^ MKs can be generated per spinner flask BR in cell-OA differentiation, which corresponds to 3.9 ± 1.0 MKs per input iPSC (Fig. 3e,f). The amount of total MKs and MKs per iPSC generated in cell-MC aggregate differentiation was significantly higher, with 18.7 ± 6.8 × 10^7^ MKs (p = 0.006) and 29.9 ± 10.9 MKs per input iPSC (p = 0.006) (Fig. 3e,f).

### MKs derived from iPSC in cell-OAs and cell-MC aggregates are polyploid

**Figure 4:**
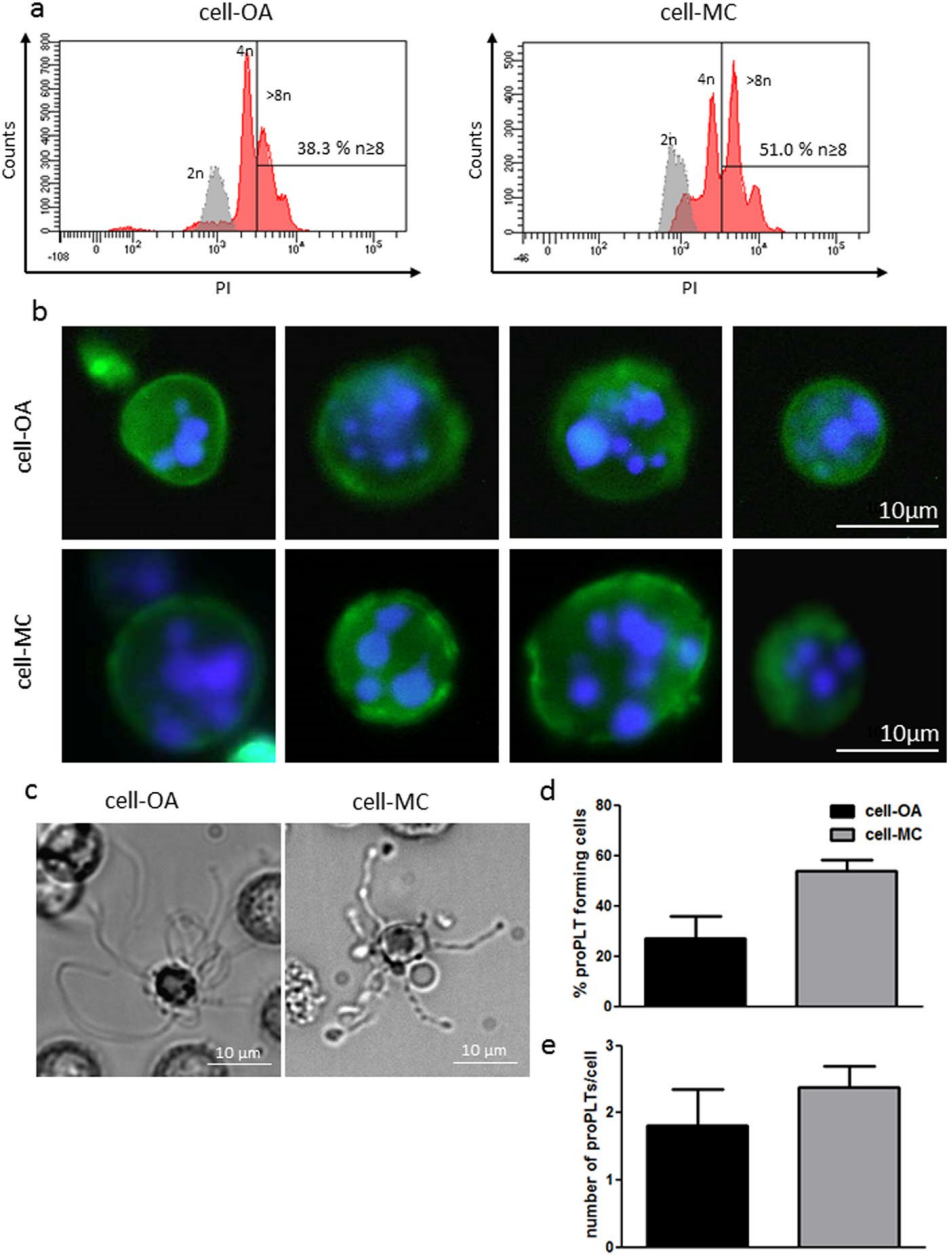
iPSC-derived MKs differentiated in stirred flasks show increased DNA content and the capability to form proPLTs. Polyploidy of the harvested cells was analyzed quantitatively by flow cytometry after propidium iodide staining. Exemplary histograms show MK DNA contents (red) and iPSC DNA content (grey) (**a**). DAPI staining revealed polyploidy in fluorescence microscopic analyses (**b**). Formation of proPLTs was shown in cells from both differentiation setups (**c**). Quantification of proPLT forming cells (**d**) and proPLTs per proPLT forming cell (**e**) were performed. Graph is depicted as mean ± SD of n = 4.

Polyploidy is a typical feature of mature MKs, which undergo endomitosis during their development^29^. Flow cytometric analysis of PI stained MKs showed that 35.0 ± 16.2% of cell-OA-derived MKs and 51.8 ± 6.1 of cell-MC aggregate derived MKs showed DNA contents higher than 8n (Fig. 4a), compared to 2n iPSCs. DAPI staining and fluorescence microscopy of cells harvested from the BR revealed a polyploid phenotype (Fig. 4b). These data indicate that MKs produced in the BR resemble bone-marrow-derived MKs.

### iPSC-MKs mature into ProPLTs

*In vivo*, the major task of MKs is to produce PLTs. Mature MKs form long protrusions known as proPLTs, from which PLTs are shed and released into the blood stream^30^. MKs were harvested from the spinner flask and seeded under static conditions for analysis. Cell-OA and cell-MC aggregate derived MKs were capable of forming proPLTs (Fig. 4c). The percentage of proPLT forming cells, as well as the number of proPLTs per proPLT forming cell, were slightly increased in MKs harvested from MC-based differentiation compared to cell-OA differentiation (Fig. 4e,f). These data further indicate that MKs produced in cell-OAs and cell-MC aggregates are functional.

### Impact of irradiation on proPLT development

**Figure 5:**
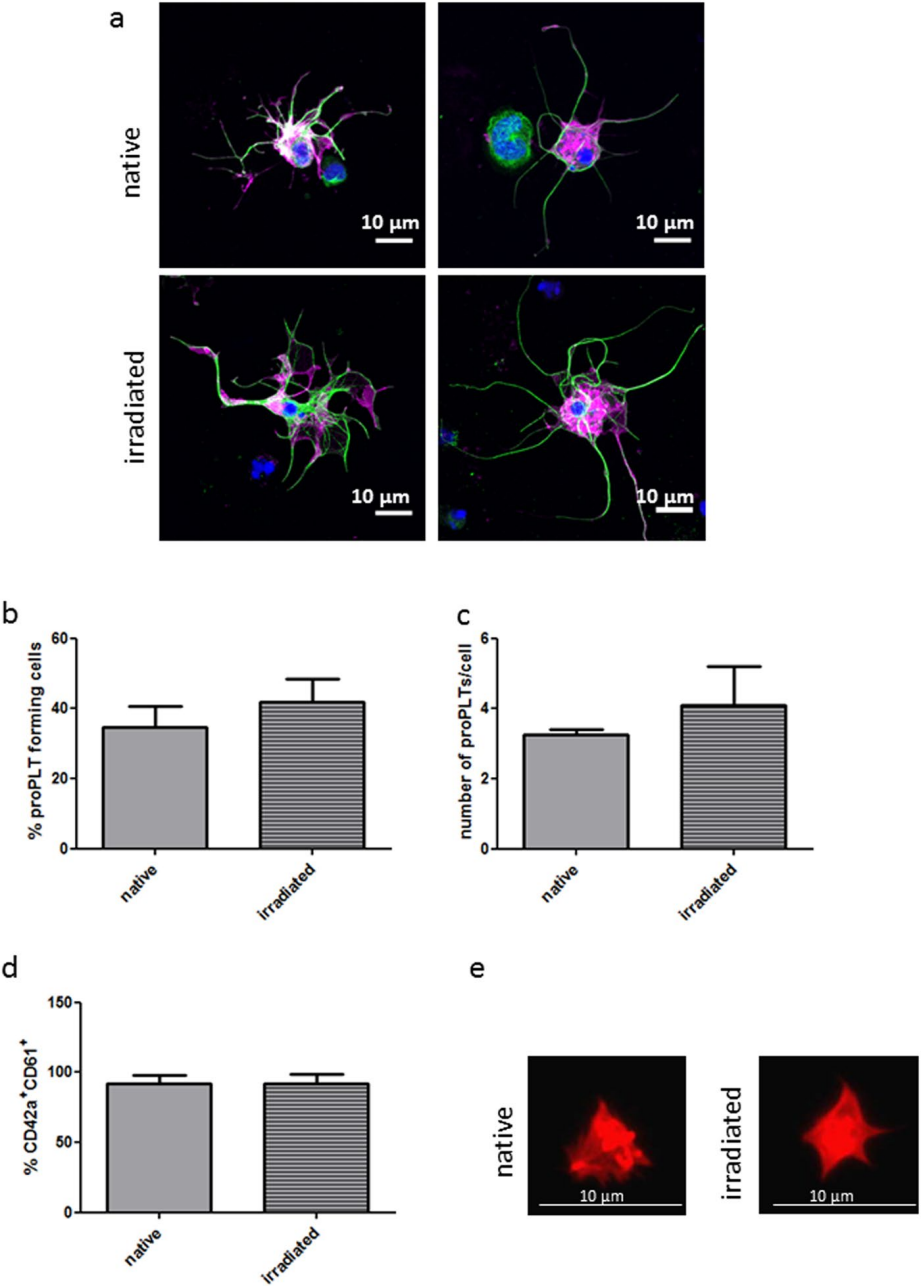
Irradiation does not affect proPLT and PLT formation from iPSC-derived megakaryocytes produced in cell-MC differentiation. MKs produced from iPSCs in stirred cell-MC cultivation, both non-treated (upper row) and irradiated (lower row) were seeded for 24 h on poly-l-lysine-coated coverslips. Representative immunofluorescence images of cells labelled for β-tubulin (green) and CD61 (magenta) show comparable proPLT structures between the two conditions (**a**). At least ten images were analyzed and the frequency of proPLT forming cells (**b**) or the number of proPLTs per proPLT forming cell (**c**) was not affected by irradiation. One day after irradiation, PLTs produced *in vitro* from cell-MC MKs were identified by size and CD41 expression by flow cytometry and further analyzed for the co-expression of CD42a and CD61. No difference between non-treated and irradiated cells was detectable (**d**). PLTs produced *in vitro* from non-treated and irradiated MKs were both able to adhere to fibrinogen-coated surfaces (**e**). Graphs are depicted as mean ± SD of n = 3 in (**b**,**c**) and n = 7 in (**d**).

Non-treated and irradiated MKs from MC-based differentiation showed proPLT formation with β-tubulin rich structures (Fig. 5a). Both the frequency of proPLT forming cells and the number of proPLTs per cell were comparable in the two conditions, or slightly increased after irradiation (Fig. 5b,c).

Furthermore, the PLTs produced by MKs from the MC-based differentiation were harvested one day after irradiation and compared to PLTs produced by non-treated MKs. A gating strategy based on size and CD41 expression was used to identify PLT-like particles via flow cytometry. These PLT-like particles were analyzed for their co-expression of CD42a and CD61. Comparable frequencies of double positive PLTs were observed, with 91.8 ± 6.3% from non-treated MKs and 91.8 ± 7.1% from irradiated MKs (Fig. 5d). In addition, both irradiated and non-irradiated PLTs adhered to fibrinogen-coated surfaces (Fig. 5e).

### MKs produced in the bioreactor are able to produce PLTs *in vivo*

**Figure 6:**
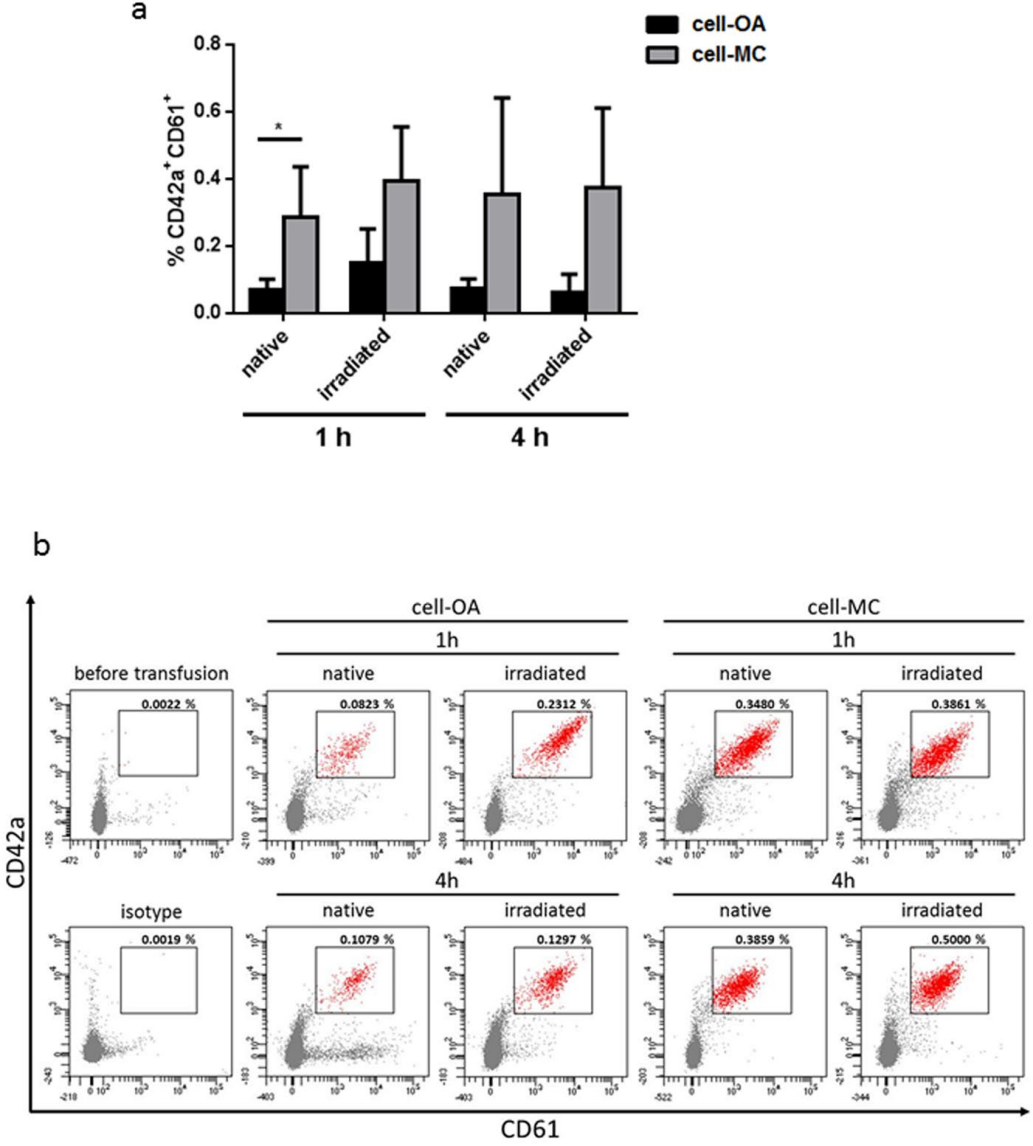
Megakaryocytes differentiated in stirred bioreactors produce PLTs after transfusion in a mouse model. MKs produced from iPSCs in stirred cell-OA or cell-MC cultivation, both non-treated and gamma-irradiated were transfused to NOD/SCID/IL-2Rγc^−/−^ mice. After 1 h and 4 h, blood was drawn and analyzed for human PLTs. The gate shows human CD42a^+^CD61^+^ cells within the mouse blood cell population. The graph (**a**) and representative plots (**b**) show percentages of CD42a^+^CD61^+^ cells. Graph is depicted as mean ± SD of n = 4.

To demonstrate the ability of the iPSC-derived MKs to produce PLTs *in vivo*, a mouse model was used. Irradiated or non-irradiated MKs were transfused to immune-deficient mice and the content of human PLTs in their blood was analyzed after 1 h and 4 h. At both time points, and in all conditions, human PLTs were detected in the murine blood (Fig. 6). In mice treated with irradiated MKs from the cell-OA differentiation, 0.1487 ± 0.1032% and 0.0620 ± 0.0543% of human PLTs were detected in the circulation 1 h and 4 h after transfusion, respectively. 0.0706 ± 0.0308% and 0.0722 ± 0.0299% of human PLTs produced from cell-OA MKs were detected 1 h and 4 h after transfusion with non-irradiated MKs, respectively (Fig. 6a, exemplary plots shown in Fig. 6b). After transfusion of MKs produced in the MC-based differentiation setup 0.2841 ± 0.1523% human PLTs and 0.3545 ± 0.2863% human PLTs were detected after 1 h and 4 h, respectively. The transfusion of irradiated MKs from the cell-MC differentiation yielded 0.3930 ± 0.1622% human PLTs and 0.3742 ± 0.2368% human PLTs after 1 h and 4 h, respectively (Fig. 6).

## Discussion

Although extensive research has been dedicated to developing processes for differentiating PLTs from MKs, no sustainable process for large-scale MK production is available. 2D culture systems are not able to fulfil the demands of high MK cell yields at low production costs^16,17^. Suspension culture systems are the most promising approaches with respect to handling, scalability, cell yields, monitoring and control of specific parameters, reproducibility, and cost-effectiveness^17^. The frequent use of stirred suspension BR systems for the industrial production of vaccines and antibodies shows their potential for economically successful process development. Cell production for advanced therapy medicinal products (ATMPs) in BRs has also been demonstrated^17,18^. In this study, we have focused on establishing a robust method for the production of iPSC-derived MKs, which can either be used to supply PLT-producing BRs or be directly used for transfusion or regenerative therapies.

Here, we demonstrate the feasibility to produce high numbers of MKs by combining cell-aggregate cultivation of iPSCs in suspension culture with MC technology in stirred spinner flasks BR. Although, we have previously demonstrated the efficient generation of other mesodermal derivatives, i.e. hPSC-derived cardiomyocytes from cell-OAs^22,31^, the presence of MCs resulted in a significantly increased yield of MK production in the current study. Non-porous polystyrene MCs were coated with LN521, which was shown to support hematopoietic differentiation^28^. Laminins are known to support cell adhesion and modulate cell differentiation, phenotype stability, and inhibit apoptosis^32^. LN521 was shown to facilitate the adhesion of a variety of cell types and to bind to polystyrene^33^. Therefore, non-porous polystyrene MCs provide a valuable growth surface in suspension BR systems. Moreover, the high surface to volume ratio of MCs permits a high cell density^15,17^, whilst the size of cell-OAs is hard to control and gradually increases. In large aggregates, the transport of nutrients and cytokines is hindered and can negatively influence cell proliferation and differentiation^16,17^. In contrast, a consistent cell-MC aggregate size with simultaneous increase of cell-MC aggregate numbers per volume, constituting a self-regulating microenvironment, was reported^34^.

In both differentiation strategies using cell-OAs and cell-MC aggregates, MKs were harvested as early as day 12 of differentiation, resembling a 2D differentiation process previously established^28^. However, during subsequent cultivation for 7 days the MK frequency in suspension increased, suggesting that the cell suspension harvested at an early state of differentiation consist partly of progenitor cells, which are further proliferating and maturating. This observation was confirmed by the expression of pluripotency and hematopoietic progenitor markers. In MC containing differentiations, slightly higher levels of CD34 and CD43 co-expression were observed compared to those detected in cell-OA differentiations. This may suggest that MCs or the laminin used for coating tend to support the induction of hematopoietic progenitors. However, both culture strategies render typical mature MKs characterized by polyploidy and the capacity to form proPLTs, an indicator of high quality and functionality. While the amount of CD41^+^CD42a^+^CD61^+^ cells increased during 7 days of continued cultivation after cell harvest on day 12 and 19 from the MC-based differentiation cultures, this effect appears in the cell-OA-based differentiation only for cells harvested on day 12. This observation suggests that the numbers of progenitors in cell-OA-cultures are exhausted earlier. The frequency of proPLT forming cells and the number of proPLTs per cell show no significant difference between both MK production setups, although values for MKs produced with the aid of MC are increased. Remarkably, the total number of MKs produced, as well as the relative yield of MKs per input iPSC, were significantly increased in MC-containing differentiation culture compared to the cell-OA approach. Hence, MC-based stirred suspension BRs showed higher efficiency for the production of large MK yields. Typically, the use of carriers may pose a safety risk to the development of cell therapeutic products as there is a need to separate the cell product from the material before application^17^. Since differentiated MKs are naturally released into the supernatant and can be easily separated by filtration, this approach is safe.

**Figure 7:**
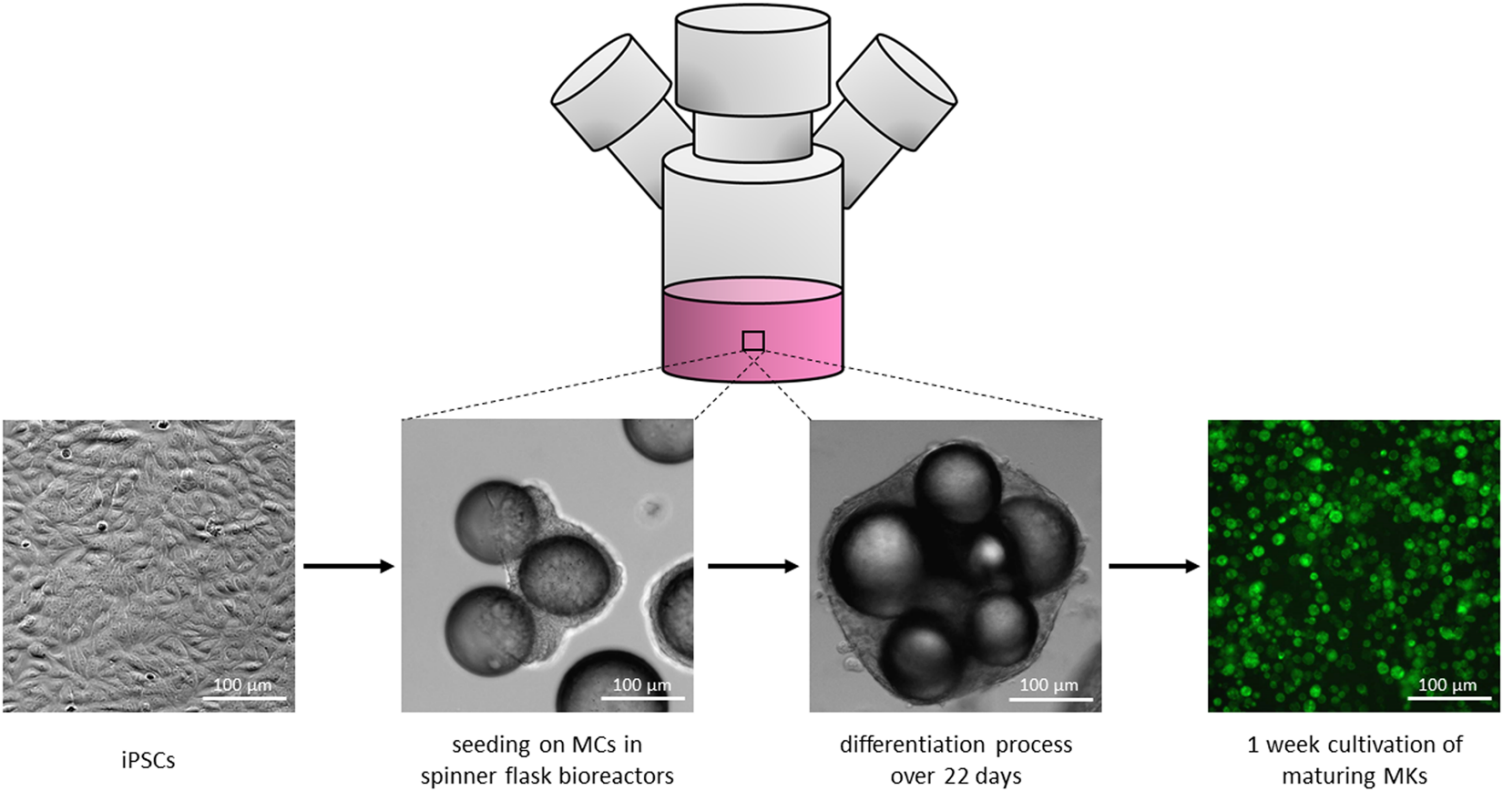
Schematic representation of the suggested process for MK production in stirred bioreactors. According to the results obtained in this study, we suggest to use MC-based iPSC-differentiation into MKs in stirred spinner flask bioreactors for future applications. During this process, iPSCs are seeded on LN521-coated MCs. During 22 days of differentiation in stirred suspension systems, large numbers of MKs are produced and can be easily harvested from the supernatant. After subsequent cultivation and maturation the MKs can be used for PLT production or as a PLT substitute in transfusion.

Using a previously established mouse model, iPSC-derived MKs showed the capability to produce PLTs, emphasizing their potential as PLT transfusion surrogates. As MC-based cultivation methods showed higher MK yields compared to cell-OA, we suggest using MC-based differentiation for future large-scale MK production (Fig. 7).

As differentiation protocols typically do not lead to pure populations of the target cells, the characterization of potential “contaminating” blood cells was performed. The presence of other cell types such as T-cells, erythrocytes, and myeloid cells was demonstrated to be lower than 3% for each lineage in our suspension cultures (Supplemental Fig. 2a). However, expression of progenitor markers was detected. Approximately 25% of the cells expressed CD71, a marker of erythroid precursors^35^. This observation tallies with the tight association of MK and erythroid lineages, which are both developed from the common megakaryocyte-erythroid-progenitor^36^. CD36 was expressed by 40–50% of the harvested cells (Supplemental Fig. 2a). While CD36, a scavenger receptor protein, is regularly associated with the uptake of long fatty acids into muscle or adipose tissue^37^, it is also expressed in megakaryocytic progenitors^38^. Moreover, the hematopoietic progenitor cell marker CD45 was expressed in more than 60% of the harvested cells, indicating incomplete MK maturation^38^. Further analysis of the co-expression of CD36, CD45, and CD71 together with typical MK markers CD41 and CD42a in MKs harvested from MC-based differentiation, revealed that all three markers are expressed higher in CD41^+^CD42a^+^ cells than in CD41^−^CD42a^−^ cells, confirming their hematological origin.

Notably, SSEA4 expression decreased to about 3% in cell-OA differentiation and to lower than 1% in MC-based differentiation, suggesting a loss of pluripotency in the suspension cultures. Nevertheless, extreme caution is required for the preparation of iPSC-derived therapeutic products to prevent teratoma formation. Numerous procedures to diminish contamination with residual pluripotent cells have been established, which include the combination of cell sorting and specific cytotoxic antibodies^39^. Further, PLT preparations for transfusion purposes can be irradiated without loss of PLT quality to prevent the co-transfusion of proliferating cells^40^. Interestingly, mature MKs, which have terminated cell cycling are also not affected by irradiation with respect to their functional properties^41^. Neither the frequency of proPLT forming cells, nor the number of proPLTs per cell was negatively affected by irradiation. In addition, the β-tubulin structures were also not affected by irradiation. Remarkably, the ability of irradiated MKs to produce PLTs in mouse circulation was not impaired. Hence, gamma-irradiation is an easy and quick method to achieve a high safety level and reduce the cancerogenic risk of iPSC-derived MKs.

*In vitro* produced PLTs constitute an analogous surrogate for donated PLTs and are available for coagulation immediately after transfusion. However, the production of PLTs is complex as numerous factors play a role in their development^8^. Furthermore, the preservation of a quiescent state and ability to be activated is critical. Even the quality of donor PLTs suffers from decay and activity loss due to handling and storage^2^. In contrast, *in vitro* produced MKs and progenitors can be stored frozen^12,13,42^, and MKs can produce PLTs within time frames as short as one hour^28^. As these PLTs would be freshly produced, instead of being collected over an expanded time frame, a longer life time in the patient’s circulation could be expected. Furthermore, the first clinical trials using CD34^+^ cell-derived MKs after high dose chemotherapy showed promising results in terms of safety in long term follow up and a reduced need for PLT transfusion^3–5^. According to Xi *et al*., 11 × 10^6^ MKs/kg body weight or 66 × 10^7^ MKs per 60 kg patient per transfusion is required^3^. With MC-based MK differentiation from iPSCs, this amount of cells can easily be achieved in as little as 200 ml of differentiation culture in stirred suspension BRs. Furthermore, the regenerative potential of MKs is of extreme interest. It was shown that MKs can promote hematopoietic stem cell (HSC) proliferation *in vitro*^43^ and *in vivo*^44^ after chemotherapy. *In vitro* produced MKs therefore have the potential to support HSC expansion and early PLT production after stem cell transplantation. Thus, the availability of large-numbers of MKs would facilitate the development of innovative MK-based therapeutic strategies.

Altogether, we have presented a stabile platform to produce large numbers of MKs, with reduced work-load, handling steps, and material costs. This system is easily scalable and can be adapted to automatization and online control. This study has the potential to bring the robust and standardized use of *in vitro* manufactured MKs and PLTs one step closer to reality.

## Methods

### Cultivation of iPSCs

The human iPSC line hCBiPSC2 (kindly provided by Ulrich Martin) was derived from human cord blood endothelial cells^45^ and maintained in feeder- and xeno-free conditions as previously described^28^. Briefly, the cells were seeded with a density of 50.000 cells/cm^2^ on 12 well non-treated culture plates (Falcon by Corning, Corning, USA) coated with LN521 (Biolamina, Sundbyberg, Sweden). iPSCs were fed daily with StemMACS iPSC brew XF, human medium (Miltenyi Biotec, Bergisch Gladbach, Germany) and passaged 2–3 times per week, when confluency was reached.

### Differentiation of iPSCs to MKs

Differentiation of iPSCs to MKs in suspension was performed using the same composition of media and cytokines throughout the differentiation process as previously described for monolayer cultivation^28^. Briefly, iPSCs were seeded in StemMACS iPSC brew XF, human medium. Afterwards, APEL 2 medium (StemCell, Vancouver, Canada) supplemented with 5% Protein-free Hybridoma Medium II (PFHMII, Gibco by Life Technologies, Darmstadt, Germany) and cytokines was added. On day 0 and 2, BMP4 and VEGF (50 ng/ml) were added to the medium, on day 4 and 8 SCF, TPO (50 ng/ml), and IL-3 (25 ng/ml) (all cytokines from Peprotech, Hamburg, Germany) were used, and from day 12 on medium containing SCF and TPO (50 ng/ml) was exchanged two times per week. Two different approaches of scalable suspension culture were performed, each in 50 ml medium in spinner flasks bioreactors (Pfeiffer Electronic GmbH, Greifenstein, Germany). At the start of differentiation, cells were dissociated to single cell suspension and seeded in the flasks. In the case of cell-OA differentiation, Rho-associated protein kinase (ROCK) inhibitor Y-27632 (10 ng/ml, Calbiochem Novabiochem GmbH, Sandhausen, Germany) was used to help with cell survival until cell-OAs were formed under agitation (50 rpm), with 12.5 × 10^6^ cells seeded per 50 ml. For MC-based cultivation serum and animal product free, biocompatible, non-porous polystyrene SoloHill plastic microcarriers (MC, PALL, Dreieich, Germany, 281.25 mg per 50 ml) with a size distribution of 125–212 µm and absent absorption of toxic side products were used. With a specific weight of 1.034–1.046 g/ml the MCs can be held in suspension at minimal agitation speed^15^. Beforehand, the heat tolerant MCs were sterilized by autoclaving and coated with LN521 (Biolamina) at 4 °C for at least 24 h under agitation. LN521 was produced under chemically defined and xeno-free conditions in a HEC-cell line. 6.25 × 10^6^ iPSCs were seeded per 50 ml and after a static phase of ~24 h, which allows cell attachment and formation of cell-MC aggregates, the suspension was stirred at 25 rpm.

In both differentiation protocols, the produced MKs were harvested from the supernatant twice a week starting from day 12 and further cultivated in suspension flasks.

### Phenotype analysis of iPSC-derived MKs and PLTs

The content of MKs in the differentiation cultures was analyzed as described before^28^. Briefly, the harvested cells were stained using anti-CD41-APC/Cy7 or -FITC, anti-CD61-APC (all Biolegend, San Diego, USA), and anti-CD42a-PE (BD Biosciences, Heidelberg, Germany) antibodies. Total cell counts in the differentiation culture were calculated using a Neubauer chamber (Marienfeld Superior, Lauda Königshofen, Germany). Absolute MK counts were estimated by multiplying the total cell number by the percentage of CD41^+^CD42a^+^CD61^+^ cells detected by flow cytometric analyses. For total MK numbers, the values measured either on the day of harvest or on 3–4 or 7 days later from the cell population harvested on day 12, day 15, day 19, and day 22 were summarized. Furthermore, the cells were stained with anti SSEA4-Alexa Fluor® 647, anti-CD43-PE/Cy7 (both Biolegend), and anti-CD34-PE (BD Biosciences). IPSC-derived PLTs were separated from the MK suspension via centrifugation and stained equally for CD41, CD42a, and CD61. For all flow cytometric analyses, FACS Canto II systems and FACSDiva Software v8.0.1 were used (BD Biosciences).

### Analysis of iPSC-derived MK polyploidy

Polyploidy of the MKs was analyzed by two different methods. Qualitative analysis was performed by microscopy, while flow cytometry was used for quantitative analysis. Briefly, cells were stained with anti-CD61-FITC antibody (BD Biosciences) and 4′,6-diamidino-2-phenylindole (DAPI) nucleic acid stain (Invitrogen, Karlsruhe, Germany) for fluorescence microscopy with an Olympus IX81 microscope (Olympus, Hamburg, Germany) and analysis using the Xcellence Pro image software (Olympus). For flow cytometric analysis, cells were stained with anti-CD41-APC/Cy7 antibody (Biolegend), fixed and permeabilized with Cytofix/Cytoperm (BD Biosciences) and stained with propidium iodide (PI, Sigma-Aldrich, München, Germany) for 30 min at 4 °C in presence of RNase. The stained cells were analyzed for PI content using flow cytometry. IPSCs were used as a control.

### Assessment of proPLT formation from iPSC-derived MKs

For Brightfield analysis of proPLT formation, iPSC-derived MKs were cultured for 2 days in differentiation medium under static conditions to detect proPLT formation using an Olympus IX81 microscope combined with a digital B/W camera. Analysis was performed using the Xcellence Pro image software (Olympus).

For β-tubulin staining, MKs and proPLTs were fixed on coverslips. Coverslips (15 mm diameter, 1.5 thickness) were washed in 1 M HCl, 100% ethanol and then coated with poly-l-lysine (Sigma-Aldrich) as per the manufacturer’s instructions. Cells were seeded onto the coated coverslips in a 24 well plate and incubated for 24 h at 37 °C. Samples were fixed in 4% PFA for 15 min, washed twice with 1X PBS, permeabilized with 0.1% Triton X-100 for 5 min and blocked for in 2% BSA/PBS for 1 h at room temperature. To visualize microtubules, cells were labelled with a mouse monoclonal IgG_1_ anti-β Tubulin primary antibody (Santa Cruz, Heidelberg, Germany), washed twice with 1X PBS and incubated in a goat-anti-mouse IgG_1_ Alexa Fluor® 488 secondary antibody (Jackson ImmunoResearch, West Grove, USA). Samples were washed twice with 1X PBS and blocked for 30 min in a 5% normal mouse serum/PBS solution. CD61 was subsequently labelled with an Alexa Fluor® 647 conjugated anti-CD61 antibody (Biolegend) and nuclei were counterstained with DAPI (Invitrogen). Washed coverslips were mounted in ProLong^TM^ Gold Antifade Mountant. Samples were imaged with the Leica TCS SP8 confocal laser scanning microscope. For quantification, 10 regions were imaged per condition with the 40X/1.1 NA water immersion objective, with a z-interval of 0.35 µm. Representative images of each condition were imaged with the 63X/1.4 NA oil immersion objective, with a z-interval of 0.35 µm. Maximum intensity projections were produced with the Leica LAS X software.

For quantification, at least 10 images were analyzed. With Brightfield images, the number of proPLT forming cells from all cells was determined. For β-tubulin stained samples, the number of proPLT forming cells of the CD61^+^ cells was quantified. In both conditions, the number of proPLTs per proPLT forming cell was analyzed.

### Irradiation of iPSC-derived MKs

To assess whether gamma-irradiation mature MKs maintain the capacity to produce proPLTs and PLTs, MKs were irradiated with 30 Gy one day before analysis or *in vivo* injection.

### Attachment to fibrinogen and morphological changes of in vitro produced PLTs

The feasibility to adhere to blood coagulation related stimuli and to accordingly change morphology was assessed with a method designed according to a protocol used by Cho *et al*.^46^. 8 well PCA chamber slides (Sarstedt, Nümbrecht, Germany) were coated over night at 4 °C with 100 µg/ml fibrinogen (Sigma-Aldrich). Platelets were transferred to the coated wells and incubated in the presence of 1 mM ADP and 1 U/ml thrombin (both Sigma-Aldrich) for 90 min at 37 °C. After washing three times with PBS, the PLTs were fixated with Cytofix (BD Biosciences) for 10 min at 37 °C and washed twice with 100 mM glycine (Carl Roth, Karlsruhe, Germany) and once with PBS. The PLTs were permeabilized for 3 min with 0.2% Triton-X-100 (Sigma-Aldrich) at RT, washed three times with PBS and subsequently stained for 20 min at 37 °C with phalloidin-labelled in TexasRed (Invitrogen) diluted 1/70. The cells were washed three times with PBS and once with water. The grid of the PCA slide was detached, mounting solution with DAPI (Dianova, Hamburg, Germany) was applied and the slide was covered with a cover slip. Adhesion of PLTs to fibrinogen was analyzed by fluorescence microscopy using an Olympus IX81 microscope (Olympus) combined with a digital B/W camera (Olympus) and analyzed with Xcellence Pro image software (Olympus).

### Mouse model for PLT production *in vivo*

MKs harvested from differentiation in cell-OAs and cell-MCs were irradiated on day 18 or not. On day 19.3 × 10^6^ MKs were transfused into 8- to 12-week-old NOD/SCID/IL-2Rγc^−/−^ mice via intravenous injection into the tail vein. After 1 h and 4 h, peripheral blood was drawn from the mice and analyzed by flow cytometry for human PLTs using anti-CD61-APC (Biolegend) and anti-CD42a-PE (BD Biosciences) antibodies.

### Statistical analysis

All experiments are displayed as mean with standard deviation. The statistical analysis was done using Mann-Whitney Test run on GraphPad Prism 7 software (GraphPad Software, La Jolla, USA). The significance levels are displayed as p value (*p ≤ 0.05, **p ≤ 0.01, ***p ≤ 0.001).

### Data availability

The datasets generated during and/or analyzed during the current study are available from the corresponding author on reasonable request

## Electronic supplementary material

Supplementary Information
