# Supplementary material for: Large-scale production of megakaryocytes in microcarrier-supported stirred suspension bioreactors
Source: Sci Rep. 2018 Jul 5;8:10146. doi: 10.1038/s41598-018-28459-x (PMC6033877; doi:10.1038/s41598-018-28459-x)
Supplement: Supplementary file 1 — Supplementary Information [file 41598_2018_28459_MOESM1_ESM.pdf]

Large-scale production of megakaryocytes in microcarrier-supported stirred suspension bioreactors

Dorothee Eicke[1,4], Anja Baigger[1], Kai Schulze[2], Sharissa L. Latham[3], Caroline Halloin[4,5], Robert Zweigerdt[4,5], Carlos A. Guzman[2,5], Rainer Blasczyk[1,5] and Constança Figueiredo[1,5*]

1. Institute for Transfusion Medicine, Hannover Medical School, Hannover 30625, Germany

2. Department of Vaccinology and Applied Microbiology, Helmholtz Centre for Infection Research, Braunschweig 38124, Germany

3 Institute for Biophysical Chemistry, Hannover Medical School, Hannover 30625, Germany

4. Leibniz Research Laboratories for Biotechnology and Artificial Organs (LEBAO), Hannover 30625, Germany

5. REBIRTH Cluster of Excellence, Hannover Medical School, Hannover 30625, Germany

*Corresponding author: Dr. Constança Figueiredo

Institute for Transfusion Medicine

Carl-Neuberg-Str. 1

D-30625 Hannover, Germany

Phone: +49 511 532 9711

Fax: +49 511 532 9701

Email: Figueiredo.Constanca@mh-hannover,de

Supplementary Information

Material and Methods

Parameter analysis

On all feeding events, cell culture medium was collected and analyzed. pH values were measured using the inolab pH level 1 (WTW-Xylem, Weilheim, Germany). Levels of glucose and lactate were assessed using colorimetric assay kits according to manufacturer's instructions (Glucose Assay Kit and Lactate Assay Kit, both from Cell Biolabs, San Diego, USA).

Estimation of "contaminating" cells in the bioreactor differentiation cultures

As the differentiation efficiencies of MKs from iPSCs are lower than 100 %, differentiation cultures were also analyzed for the presence of cells from other hematopoietic lineages ("contaminating" cells). Hence, cells harvested from the differentiation culture at day 15, were stained on day 19 or day 22 with fluorochrome conjugated antibodies prior flow cytometric analyses. Cells were stained with anti-human CD3-FITC (Miltenyi Biotec) for T-cell identification, CD14-PE and CD33-FITC for myeloid lineage, CD45-APC and CD36-APC/Cy7 for hematopoietic progenitors, and CD71-APC and CD235a-PE for erythrocytes and progenitors (all from Biolegend). Furthermore a co-staining of CD36, CD45, and CD71 with CD41 and CD42a was performed on MKs harvested for the MC-based differentiation.

Results

Culture conditions during differentiation in stirred spinner flask bioreactors

The differentiation medium was analyzed for pH values, glucose levels and lactate levels in every medium change throughout the differentiation using MCs. The pH value decreased from 7.7 ± 0.0 on day 2 to values between 7.2 and 7.4 from day 4 to day 22 (Supplemental Figure 1a). Glucose levels during the differentiation ranged from 5 to 7 mM on days 12 to 22

and up to about 13 mM on day 2, while the fresh medium contained 20 mM glucose. In fresh medium lactate was not detectable. At day 2 only 3.7 ±1.2 mM lactate were reached and increased until day 6 to 9.3 ± 0.3 mM. After a decline to 6.4 ± 1.6 mM on day 8, the level remained stable in later differentiation phase.

Characterization of frequencies of “contaminating” cells in the differentiation outcome

As cell suspensions harvested form the differentiation process are not exclusively mature MKs, the frequencies of “contaminating” cells including progenitor cells and other blood cells were analyzed on day 19 or day 22 (Supplemental Figure 2). Very low frequencies of T-cells, erythrocytes and myeloid cells were present in the supernatant harvested from cell-OA and cell-MC aggregate differentiation. Marker for hematopoietic progenitor cells CD71, CD45, and CD36 were detected at higher frequencies: 25.6 ± 9.6 % and 24.9 ± 8.1 %, 63.0 ± 14.8 % and 64.8 ± 7.0 %, 40.7 ± 10.5 % and 50.4 ± 4.1 % in cells collected from cell-OA and cell-MC aggregate differentiation supernatant, respectively (Supplemental Figure 2a). The expression of CD36, CD45, and CD71 was increased in $CD41^{+}CD42a^{+}$ cells harvested form MC-based differentiation cultures compared to $CD41^{-}CD42a^{-}$ cells (Supplemental Figure 2b). The increase of CD71 is not significant, while CD36 and CD45 both were increased significantly (both $p = 0.0159$).

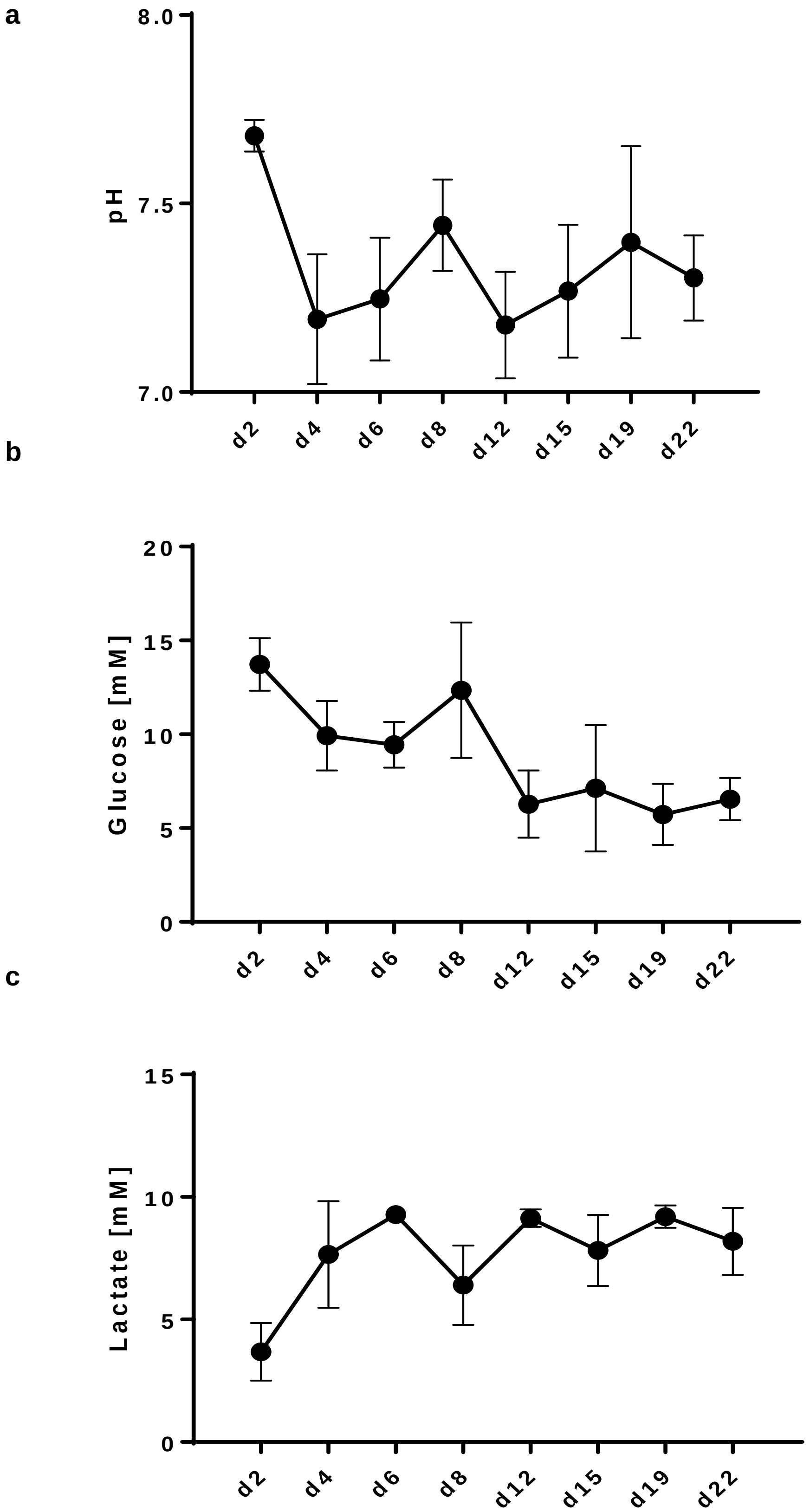

Supplemental Figure 1. Parameter variation during culture conditions. On the days of medium changes, the differentiation supernatant was analyzed for pH (a), glucose level (b), and lactate level (c). Graphs are depicted as mean ± SD of n ≥ 2.

a

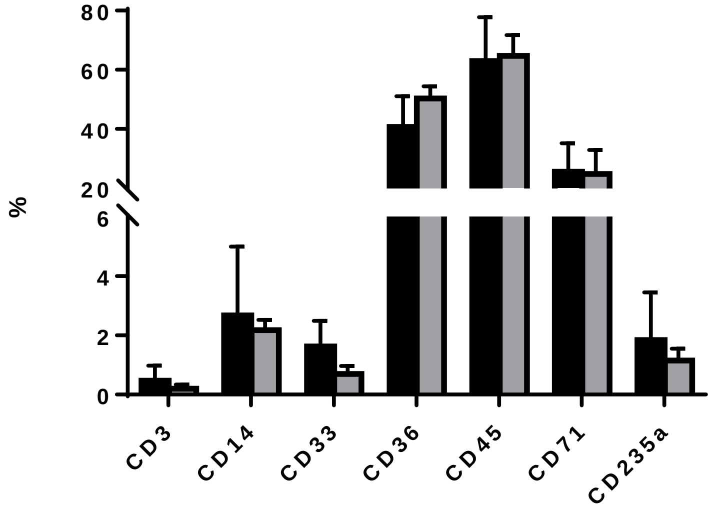

b

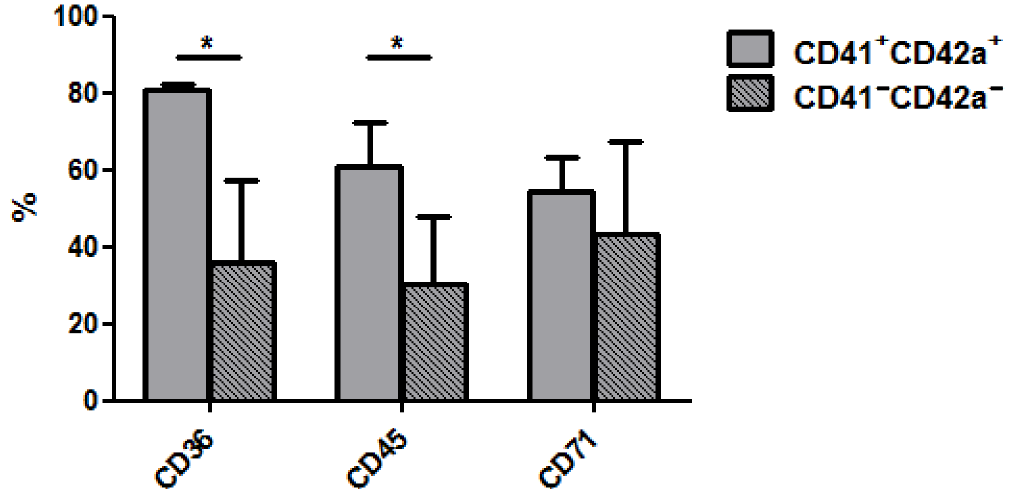

Supplemental Figure 2. Frequencies of "contaminating" cells on the differentiation cultures. Supernatants of MK differentiation culture in stirred bioreactors were analyzed for the frequency of hematopoietic progenitor cells (CD36, CD45 and CD71) as well as other "contaminating" cells such as T-cells (CD3), myeloid cells (CD14, CD33), and erythrocytes (CD235a) (a). A co-staining of CD36, CD45, and CD71 with typical MK markers CD41 and CD42a of cells resulting from MC-based differentiation was performed (b). Graphs are depicted as mean ± SD of n ≥ 4.
